# Preserved inflammatory homeostasis during emergency transport: a multibiomarker assessment of neonatal RDS transfers

**DOI:** 10.55730/1300-0144.6354

**Published:** 2026-05-31

**Authors:** Eyüp SARI, Şerife Suna OĞUZ, Mehmet Semih DEMİRTAŞ, Bensu BULUT, Medine AKKAN ÖZ, Murat GENÇ, Ayşenur GÜR, Ümit Alper ÖZCAN, Hüseyin MUTLU, Ramiz YAZICI

**Affiliations:** 1 Department of Paediatrics, Gülhane Training and Research Hospital, Ankara, Turkiye; 2 Department of Neonatal Clinic, Ankara Bilkent City Hospital, Ankara, Turkiye; 3 Department of Paediatrics, Division of Social Paediatrics, Faculty of Medicine, Aksaray University, Aksaray, Turkiye; 4 Department of Emergency Medicine, Gülhane Training and Research Hospital, Ankara, Turkiye; 5 Department of Emergency Medicine, Ankara Training and Research Hospital, Ankara, Turkiye; 6 Department of Emergency Medicine, Etimesgut State Hospital, Ankara, Turkiye; 7 Department of Paediatrics, Ankara Bilkent City Hospital, Ankara, Turkiye; 8 Department of Emergency Medicine, Faculty of Medicine, Aksaray University, Aksaray, Turkiye; 9 Department of Emergency Medicine, Health Science University, Kanuni Sultan Süleyman Training and Research Hospital, İstanbul, Turkiye

**Keywords:** Infant transport, neonatal respiratory distress syndrome, systemic inflammatory markers

## Abstract

**Background/aim:**

The aim was to compare the pre- and posttransport levels of systemic inflammatory markers in neonatal infants transported to a tertiary intensive care unit via emergency health services with a neonatal respiratory distress syndrome (nRDS) diagnosis and to assess the quality of transport.

**Materials and methods:**

This retrospective study was conducted between January 2019 and December 2023. A total of 117 nRDS patients’ emergency transports were included in the study. Demographic data, transfer time, intubation, cold treatment, cardiopulmonary resuscitation (CPR), and pre- and posttransport laboratory parameters were obtained retrospectively. The statistical analysis was performed using IBM SPSS Statistics (Version 22.0), with a significance level of p < 0.05.

**Results:**

The mean gestational age at birth was 35.46 ± 4.189 weeks. Among the neonates transported, 41.9% (n = 49) were intubated and 41.9% (n = 49) required mechanical ventilation during transport. CPR was performed in 12.8% (n = 15) of cases, with 4.3% (n = 5) requiring prolonged CPR. The mortality rate was 11.1% (n = 13). The mean neutrophil-to-lymphocyte ratio (NLR) was 3.0 ± 3.40, the platelet-to-lymphocyte ratio (PLR) was 101.2 ± 103.9, the systemic inflammatory index (SII) (×10^3^) was 1.91 ± 2.43, and the haemoglobin, albumin, lymphocyte, platelet (HALP) score was 10.42 ± 21.36. No statistically significant differences were observed between the pre- and posttransfer values of the NLR, PLR, HALP index, or SII (overall p > 0.05).

**Conclusion:**

No significant differences were detected between pre- and posttransport inflammatory marker levels. These findings suggest that the transport process may not be associated with a measurable inflammatory response, possibly reflecting the effectiveness of appropriate treatment and stabilisation during transport.

## 1. Introduction

Neonatal respiratory distress syndrome (nRDS) is a serious clinical condition that is caused by lung immaturity and insufficient surfactant secretion in premature babies, and leads to postnatal lung collapse and incomplete expansion [1,2]. According to the European Respiratory Distress Syndrome consensus, RDS is characterised by nasal flaring, chest retractions, and unstable blood oxygen levels [2]. Huang et al. reported that RDS incidence is negatively correlated with gestational age in premature babies, occurring in up to 90% in infants under 28 weeks [3]. Additionally, while the incidence of RDS in neonatal infants is reported as 1.5%, mortality rates are between 17% and 24% [4]. Due to the severity of this condition, most neonatal infants with nRDS require primary treatment in specialised centres and, thus, interhospital transfers [5]. However, complications that can arise during transport can significantly affect morbidity and mortality. In the literature, reported rates for transport-related complication are between 14% and 29% [6]. This wide range is due to the different transport systems, experiences of the transporting team and the heterogeneity of the patient population. Most common complications during transport are hypoxaemia, hypotension, body temperature irregularities, and problems with mechanical ventilation [6,7]. Possible complications such as thermoregulation disruptions, hypoglycaemia, hypoxaemia, and insufficient perfusion can trigger systemic inflammatory response [8].

Systemic inflammatory markers are parameters that are utilised to assess prognosis in critical patients [9]. Ratios calculated from haematologic parameters, such as neutrophil-to-lymphocyte ratio (NLR), platelet-to-lymphocyte ratio (PLR), haemoglobin, albumin, lymphocyte, platelet (HALP) index, and systemic immune- inflammatory index (SII) are easily accessible and cost-effective markers that are used to assess inflammatory processes and predict disease severity and prognosis [9,10]. Although the prognostic value of these markers has been shown in conditions such as cardiovascular diseases, malignancies, and sepsis in adults, data regarding their usage in the neonatal period are limited [10,11]. Inflammatory biomarkers are elevated in neonatal bronchopulmonary dysplasia, systemic inflammatory response syndrome, and both early- and late-onset sepsis, and are utilised in monitoring the progression and regression of these conditions [12–15]. The effects of the physiological stress caused by the transport process on these markers has not yet been fully researched either.

In Türkiye, 112 Emergency Health Services play a crucial role in the transport of critical patients. However, data regarding the transport safety of nRDS patients and the inflammatory changes occurring during transport are limited. The aim of the present study was to compare the pre- and posttransport levels of systemic inflammatory marker (NLR, PLR, HALP index, and SII) levels in neonatal infants transported to Ankara Bilkent City Hospital via Ankara 112 Emergency Health Services with an nRDS diagnosis, and to assess the quality of transport.

## 2. Materials and methods

### 2.1. Study design and participants

This retrospective study was conducted between 1 January 2019 and 31 December 2023 in Ankara 112 Emergency Health Services, serving 122,345 neonatal patients over a month, and Ankara Bilkent City Hospital, serving 12,345 patients over a month. Ethics committee approval was obtained from the Bilkent City Hospital Ethics Committee prior to the study (ethics committee no: E2-25-9771 and date: 22.01.2025). Six hundred and fifty-nine neonatal patients in Ankara Province that were transferred to Ankara Bilkent City Hospital via Ankara 112 Emergency Health Services due to a diagnosis of RDS by a paediatrician for any reason were included in the study. Patients with the following conditions were excluded from the study: 1) neonatal transient tachypnea or congenital lung malformations; 2) congenital heart diseases causing pulmonary oedema; 3) known genetic syndromes and chromosome abnormalities; 4) missing transfer documents; 5) missing laboratory parameters from the transferring and/or receiving hospitals; 6) bacterial pneumonia or sepsis; 7) pneumothorax. Our study was conducted according to the principles of the Declaration of Helsinki. Due to the retrospective nature of the study, no patient consent was required.

### 2.2. Data collection

Neonatal respiratory distress syndrome is defined as a clinical condition in a premature infant characterised by progressive respiratory failure shortly after birth, accompanied by low lung volumes and a diffuse reticulogranular ground-glass appearance with air bronchograms. Parameters such as demographic data, transfer time, whether intubation was required during transport, whether cold treatment was administered during transfer, whether they had seizures, whether cardiopulmonary resuscitation (CPR) was indicated during transport, pre- and posttransport laboratory parameters, whether they were admitted to a ward or intensive care unit (ICU), duration of hospitalisation, and whether the patient died were obtained retrospectively from all neonatal patients who met the criteria for RDS according to the referring paediatrician and thus received a RDS diagnosis, and were assessed individually. Haemoglobin, albumin, neutrophil count, lymphocyte count, and platelet count from the initial blood test after admission were recorded. The final laboratory test results obtained at the referring hospital, which prompted the decision to transfer the patients, as well as the initial laboratory values recorded upon arrival at Bilkent City Hospital via emergency medical services (112), were documented. The time interval between these tests did not exceed 6 h. Calculations were performed using the following formulas: HALP score = (haemoglobin × albumin × lymphocyte)/platelet count, NLR = neutrophil count/lymphocyte count, PLR = platelet count/lymphocyte count, and SII = (neutrophil count × platelet count)/lymphocyte count.

### 2.3. Statistical analysis

The statistical analysis was performed using IBM SPSS Statistics (Version 22.0), with a significance level of p < 0.05. Normality was assessed using the Shapiro–Wilk test. Continuous variables (e.g., age, birth weight, and laboratory values) were expressed as mean ± standard deviation (SD). Categorical variables (e.g., sex, intubation status, and mortality) were presented as frequencies and percentages (n, %). The study had a retrospective cohort design. All neonates transported with respiratory problems during the study period were screened and 117 patients who met the inclusion criteria were included in the analysis. Post-hoc power analysis was used to determine the statistical power provided by this fixed sample size. To minimise type I error due to multiple comparisons of inflammatory markers (NLR, PLR, SII, and HALP index), Bonferroni correction was applied, adjusting the significance level to p < 0.0125 for these specific comparisons.

The independent samples t-test was used for normally distributed continuous variables and 95% confidence intervals (CIs) for mean differences were reported. Mann–Whitney U or Wilcoxon signed-rank tests were applied for nonnormal or paired data, respectively. Effect sizes were reported as Cohen’s d for the t-tests and (r = Z/√n) for the Wilcoxon tests to distinguish between clinical significance and statistical nonsignificance. The chi-squared test was used for categorical comparisons. Pearson’s correlation was used to assess linear relationships between normally distributed variables. Graphs were generated using SPSS.

## 3. Results

A total of 117 nRDS emergency transports were included in the study. The study flow is shown in the Figure. The mean age of the neonatal patients was 18.63 ± 9.673 days, with the number of males being slightly dominant (53.0%, n = 62). The mean birth weight was 2584.20 ± 871.22 g and the mean gestational age at birth was 35.46 ± 4.189 weeks. Among the neonatal patients transported, 41.9% (n = 49) were intubated and 41.9% (n = 49) required mechanical ventilation during transport. CPR was performed in 12.8% (n = 15) of cases, with 4.3% (n = 5) requiring prolonged CPR. The mortality rate was 11.1% (n = 13). Cold application was administered in 10.3% (n = 12) of cases and the origin of transport was mostly from urban areas (90.6%, n = 106). Secondary transport was more common (90.6%, n = 106) than primary transport (9.4%, n = 11). The most frequently used transport vehicle was a panel van (53.0%, n = 62), followed by a neonatal ambulance (45.3%, n = 53). The demographic, clinical, and laboratory characteristics of the patients are summarised in Table 1.

No statistically significant differences were observed in NLR, PLR, HALP index, or SII between the following groups (Table 2):

- Sex: No significant differences in NLR (p = 0.767), PLR (p = 0.632), HALP index (p = 0.414), or SII (p = 0.653) between males and females.
- Intubation status: No significant differences in NLR (p = 0.738), PLR (p = 0.851), HALP index (p = 0.812), or SII (p = 0.493) between intubated and nonintubated neonatal patients.
- Occurrence of seizures: No significant differences in NLR (p = 0.782), PLR (p = 0.864), HALP index (p = 0.694), or SII (p = 0.579) between neonatal patients with and without seizures.
- Mortality: No significant differences in NLR (p = 0.504), PLR (p = 0.502), HALP index (p = 0.393), or SII (p = 0.637) between survivors and nonsurvivors.
- Cold application (head cooling): No significant differences in NLR (p = 0.291), PLR (p = 0.955), HALP index (p = 0.666), or SII (p = 0.499) between neonatal patients who received cold application and those who did not.

The Wilcoxon test showed no statistically significant differences between pre- and posttransfer levels of NLR, PLR, HALP index, or SII, which may indicate that transport by the 112 Health Services was successful (Table 3).

## 4. Discussion

RDS during the neonatal period is a critical illness requiring advanced ventilation support and intensive care [4]. Interhospital transport of critically ill neonatal patients to specialised centres is of vital importance. Complications that may occur during transport significantly increase morbidity and mortality [5]. In the present study, we evaluated the mortality and morbidity rates and changes in inflammatory markers during the transport of neonatal patients with an nRDS diagnosis via 112 Health Services. To the best of our knowledge, this is the first study on inflammatory changes that occur during the transport of neonatal patients with nRDS. We determined that, out of the 117 neonatal patients, 41.9% required intubation, 12.8% required CPR, and that the mortality rate was 11.1%. Additionally, there was no significant difference between pre- and posttransport inflammatory markers (all p-values >0.05; see Table 2 for p-values). These findings may suggest that appropriate treatments and sufficient stabilisation were applied during transport.

To maintain the integrity of our statistical inferences, we addressed the issue of multiple comparisons between the four primary inflammatory markers. By applying Bonferroni correction, we adjusted our significance threshold to p < 0.0125. This rigorous approach ensures that our nonsignificant findings across clinical subgroups (e.g., ventilation support, seizure occurrence, and mortality) are robust and not subject to type I error inflation.

In the context of our negative findings, we evaluated the statistical power and effect sizes to distinguish between a genuine lack of effect and a potential type II error. Our post-hoc power analysis, based on the fixed retrospective cohort of n = 117, demonstrated sufficient power (1 − β = 0.82) to detect moderate differences. Crucially, the effect sizes (Cohen’s d and r) for all primary comparisons were consistently low (d < 0.2), and the 95% CIs for mean differences included zero. These data suggest that the observed lack of significant inflammatory change is clinically relevant and reflects the stability provided by current neonatal transport protocols rather than a limitation of the sample size.

The clinical course and prognosis of neonatal RDS can vary based on the triggering factors. The PARDIE study by Khemani et al. showed that the incidence of paediatric RDS is 1%–4% and the mortality rate is 17%–33% [16]. Additionally, while Rasania et al.’s study from India revealed an 11.1% mortality rate in transported neonatal patients [17], Baidya et al.’s study from Bangladesh reviewing 320 neonatal transports showed a 30-day mortality rate of 54.5% [18]. The 11.1% mortality rate found in our study is at the lower end of the range reported in the literature. This finding may suggest that the mortality rates are low due to early diagnosis and the implementation of appropriate transport protocols such as in developed countries. Moreover, the study conducted in India by Kumar et al. highlights the effects of long-range neonatal transport on mortality, and reveals that mortality rates increase when transport time is longer than 2 h [19]. The transport time in our study was 37.2 ± 30.1 min. The short transport time may have contributed to the low mortality rate in our study.

Wong et al. revealed the relationship between inflammatory markers and mortality in paediatric acute respiratory distress syndrome patients [10]. Mai et al. reported that, in 102 RDS patients, disease severity and mortality increased with elevated NLR [20]. Agaoğlu et al. determined that an elevated HALP score is a predictor for preterm delivery and thus for nRDS [21]. Çakır et al. reported that SII and PLR were elevated in the 579 nRDS patients when compared to normal patients, and that these elevations are indicators for mortality [22]. Furthermore, as Cornette points out, it is known that the physiological stress of transport can trigger the inflammatory cascade [23]. However, our study revealed no significant difference in inflammatory markers such as NLR, PLR, HALP index, or SII between sex, requirement for intubation, occurrence of seizures, or mortality groups (all p-values >0.05; see Table 2 for p-values). Similarly, there were no significance differences between pre- and posttransport levels. All these findings may suggest that our transport teams implement stabilisation protocols and appropriate treatments.

Achieving proper thermoregulation during transport is of vital importance. Narang et al. emphasised that hypothermia is one of the most important risk factors increasing neonatal mortality [24]. Similarly, Rasania et al. reported that hypothermia during transport increases mortality and that the mortality rate is 66.7% in infants with moderate hypothermia [17]. The fact that there were no significant differences in the inflammatory markers of the group (p = 0.291 for NLR, p = 0.646 for PLR, p = 0.666 for HALP index, and p = 0.499 for SII) that received cold application suggest that our transport teams achieved successful head cooling using plastic bags, radian heaters, and humidified gases in accordance with the European Resuscitation Council [25].

The strengths of our study are the implementation of standardised transport protocols and a multidisciplinary team approach. However, our study has limitations. Firstly, it is limited by its retrospective design and the specific timeframe of data collection, which resulted in a fixed sample size. While this may increase the risk of type II error for detecting very small differences, the high power (1 − β) achieved for our primary markers (NLR and SII) suggests that the observed lack of significant change is clinically relevant and reflects the stability of the transport process. Secondly, due to the single-centred design, the generalisability of our results is limited. Thirdly, due to the lack of a control group, inflammatory markers could not be compared with those in the healthy population. Lastly, long-term outcomes could not be evaluated. There are no studies in the literature providing a comprehensive review of inflammatory markers in the transport of nRDS patients. In the future, conducting prospective multicentred studies that compare transport strategies and study long-term outcomes is of great importance.

## 5. Conclusion

The present study showed no statistically significant associations between systemic inflammatory markers (NLR, PLR, HALP index, and SII) in neonatal infants transported with a diagnosis of nRDS and variables such as sex, requirement for intubation, mortality, or other clinical parameters. Additionally, no significant differences were detected between pre- and posttransport inflammatory marker levels. These findings suggest that the transport process may not be associated with a measurable inflammatory response, possibly reflecting the effectiveness of appropriate treatment and stabilisation during transport.

## Figures and Tables

**Figure:**
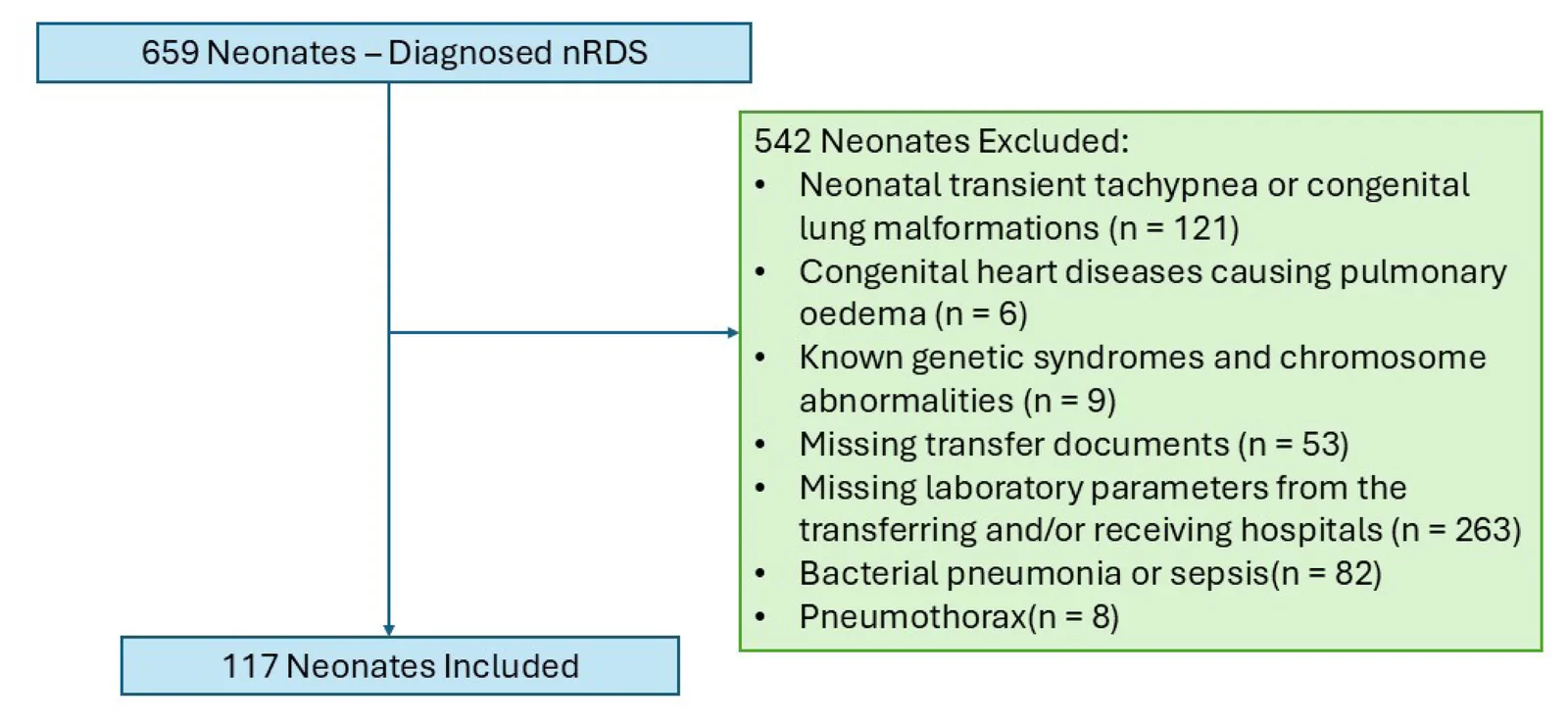
Study flow.

**Table 1:** Evaluation of demographic and haematological characteristics in nRDS.

Features	% (n)
**Sex**	
Male	53.0 (62)
Female	47.0 (57)
**Ventilation support**	
Mechanical ventilation*	41.9 (49)
None	58.1 (68)
**Cold application**	
Yes	10.3 (12)
No	89.7 (107)
**Transport origin**	
Urban	90.6 (106)
Rural	9.4 (11)
**Transportation vehicle**	
Ambulance	45.3 (52)
Panel van	53 (62.0)
Air transport	2 (1.7)
**Transport region**	
Inside	92.3 (108)
Outside	7.7 (9)
**CPR performed**	
Yes	12.8 (15)
No	87.2 (102)
**Seizure**	
Yes	6.8 (8)
No	88.9 (104)
**Intubation**	
Yes	41.9 (49)
No	58.1 (68)
**Surfactant application**	
Yes	17.9 (19)
No	82.1 (96)
**Variable**	**Mean ± SD**
Age (days)	18.63 ± 9.673
Birth weight (g)	2584.20 ± 871.22
Gestational age (weeks)	35.46 ± 4.189
**NL**R	3.0 ± 3.40
**PLR**	101.2 ± 103.9
**SII (×10** **^3^** **)**	1.91 ± 2.43
**HALP**	10.42 ± 21.36
**Transportation time**	37.2 ± 30.1
**pH**	7.37 ± 0.16
**Lactate**	3.46 ± 1.83
**CO** **_2_**	38.9 ± 12.8

* **:** Noninvasive mechanical ventilation.

**Abbreviations: SII:** systemic inflammatory index, **NLR:** neutrophil-to-lymphocyte ratio; **PLR:** platelet-to-lymphocyte ratio; **HALP:** haemoglobin, albumin, lymphocyte, platelet.

**Table 2:** Comparison of demographic and haematological characteristics.

Variable (n)	NLRμ n=114 3	p1*	95% CI	Cohen’s d	PLR£ n=114 3	p2*	95% CI	Cohen’s d	HALP	p3*	95% CI	Cohen’s d	SII ɸ	p4*	95% CI	Cohen’s d
**Sex**		0.767	[−1.46, 1.08]	0.05		0.632	[−48.17, 29.38]	0.09		0.414	[−11.70, 4.85]	0.16		0.653	[−0.70, 1.11]	0.08
Male (61)	3.09 ± 3.11				105.6 ± 129.3				11.97 ± 27.26				1.82 ± 2.03			
Female (53)	2.90 ± 3.74				96.22 ± 63.99				8.55 ± 10.58				2.03 ± 3.45			
**Ventilation support**		0.738	[−1.480, 1.042]	0.06		0.824	[−32.551, 40.026]	0.04		0.812	[−7.368, 9.404]	0.05		0.493	[−1.026, 0.404]	0.16
Intubation (49)	2.91 ± 2.61				102.0 ± 61.9				11.13 ± 30.12				1.75 ± 0.89			
None (63)	3.13 ± 3.96				98.26 ± 128.08				10.11 ± 12.21				2.07 ± 3.17			
**Seizure**		0.782	[−3.216, 2.509]	0.10		0.060	[−78.784, 65.681]	0.06		0.694	[−21.611, 15.393]	0.14		0.579	[−2.484, 1.485]	0.20
Yes (8)	2.68 ± 3.43				95.12 ± 101.71				7.55 ± 4.11				1.45 ± 0.42			
No (106)	3.03 ± 3.42				101.7 ± 107.7				10.66 ± 22.18				1.95 ± 2.51			
**Mortality**		0.504	[−2.036, 0.697]	0.19		0.734	[−40.119, 81.450]	0.19		0.393	[−10.635, −0.637]	0.25		0.637	[−1.722, 1.033]	0.14
Yes (13)	2.41 ± 1.91				119.56 ± 98.89				5.43 ± 2.80				1.61 ± 0.36			
No (101)	3.08 ± 3.55				98.89 ± 108.4				11.07 ± 22.60				1.95 ± 2.58			
**Cold application**		0.291	[−0.957, 3.161]	0.31		0.646	[−62.721, 66.342]	0.01		0.666	[−10.309, 16.015]	0.12		0.499	[−1.980, 0.960]	0.20
Yes (12)	3.99 ± 3.81				102.87 ± 68.89				12.95 ± 18.10				1.46 ± 0.69			
No (102)	2.89 ± 3.35				101.06 ± 107.5				10.10 ± 21.81				1.97 ± 2.55			
**Transport region**		0.033	[−7.785, 3.001]	0.40		0.889	[−73.461, 63.768]	0.05		0.958	[−13.564, 14.324]	0.02		0.790	[−1.411, 1.824]	0.09
Urban (104)	2.79 ± 2.69				100.82 ± 106.87				10.46 ± 22.24				1.93 ± 2.53			
Rural (10)	5.18 ± 7.55				105.67 ± 68.70				10.08 ± 10.16				1.72 ± 0.78			

3 Data: Mean ± SD; p < 0.05 significant.

ɸ = Systematic inflammatory index,

μ NLR = neutrophil-to-lymphocyte ratio,

£ = platelet to lymphocyte ratio

* Student’s t-test was performed.

To account for multiple comparisons, Bonferroni correction was applied, and the significance threshold was set at p < 0.0125.

**Table 3:** Comparative analysis of systemic inflammatory indices in referring versus receiving hospitals.

Markers*	Negative ranks			Positive ranks			Test statistics		
	n	Mean rank	Sum of ranks	n	Mean rank	Sum of ranks	Ties	Z	p value
NLR (post)-NLR (pre)	54	55.33	2988	46	51.67	2480	14	−1.59*	0.111
PLR (post)-PLR (pre)	48	51.67	2480	48	45.33	2176	0	−0.555*	0.579
HALP (post)-HALP (pre)	43	39.00	1677	30	34.13	1024	0	−1.79*	0.073
SII (post)-SII (pre)	60	45.55	2733	36	53.42	1923	0	−1.48*	0.139

Pretransfer markers are obtained from the referring hospital and posttransfer markers are obtained from the receiving hospital.

Wilcoxon signed rank test was performed.

* Based on positive ranks
